# Preclinical models of epithelial ovarian cancer: practical considerations and challenges for a meaningful application

**DOI:** 10.1007/s00018-022-04395-y

**Published:** 2022-06-16

**Authors:** Alessandra Ciucci, Marianna Buttarelli, Anna Fagotti, Giovanni Scambia, Daniela Gallo

**Affiliations:** 1 Unità di Medicina Traslazionale per la Salute della Donna e del Bambino, Dipartimento Scienze della Salute della Donna, del Bambino e di Sanità Pubblica, Fondazione Policlinico Universitario A. Gemelli, IRCCS, Largo A. Gemelli 8, 00168 Rome, Italy; 2 Dipartimento Universitario Scienze della Vita e Sanità Pubblica-Sezione di Ginecologia ed Ostetricia - Università Cattolica del Sacro Cuore, Largo A. Gemelli 8, 00168 Rome, Italy; 3 Dipartimento Scienze della Salute della Donna, del Bambino e di Sanità Pubblica, Fondazione Policlinico Universitario A. Gemelli, IRCCS, Largo A. Gemelli 8, 00168 Rome, Italy

**Keywords:** Primary EOC cells, Organoids, Patient-derived EOC explants, Patient-derived EOC xenografts, Humanized mouse models, GEMMs

## Abstract

Despite many improvements in ovarian cancer diagnosis and treatment, until now, conventional chemotherapy and new biological drugs have not been shown to cure the disease, and the overall prognosis remains poor. Over 90% of ovarian malignancies are categorized as epithelial ovarian cancers (EOC), a collection of different types of neoplasms with distinctive disease biology, response to chemotherapy, and outcome. Advances in our understanding of the histopathology and molecular features of EOC subtypes, as well as the cellular origins of these cancers, have given a boost to the development of clinically relevant experimental models. The overall goal of this review is to provide a comprehensive description of the available preclinical investigational approaches aimed at better characterizing disease development and progression and at identifying new therapeutic strategies. Systems discussed comprise monolayer (2D) and three-dimensional (3D) cultures of established and primary cancer cell lines, organoids and patient-derived explants, animal models, including carcinogen-induced, syngeneic, genetically engineered mouse, xenografts, patient-derived xenografts (PDX), humanized PDX, and the zebrafish and the laying hen models. Recent advances in tumour-on-a-chip platforms are also detailed. The critical analysis of strengths and weaknesses of each experimental model will aid in identifying opportunities to optimize their translational value.

**Supplementary Information:**

The online version contains supplementary material available at 10.1007/s00018-022-04395-y.

## Background

Worldwide, estimated 313,959 new cases of ovarian cancer (OC) and almost 207,252 cancer deaths occurred in 2020 [1]. Therefore, despite the last years have seen many important advances in OC with newly identified therapeutic opportunities, the disease remains the most deadly of all gynaecological cancers [2]. Disappointingly, long-term follow-up in the UK Collaborative Trial of Ovarian Cancer Screening (UKCTOCS) has also provided evidence that neither annual multimodal screening nor annual transvaginal ultrasound screening approach significantly reduced deaths from ovarian and tubal cancer [3].

Ovarian cancer encompasses a collection of neoplasms with distinct epidemiological and genetic risk factors, precursor lesions, patterns of spread, molecular events during oncogenesis, response to chemotherapy, and prognosis [4]. Over 90% of ovarian malignancies are categorized as epithelial ovarian cancers (EOC), and currently, five main types are identified: high-grade serous (HGSOC 70%), low-grade serous (LGSOC < 5%), mucinous (MOC 3%), endometrioid (EnOC 10%), and clear-cell (CCC 10%) carcinomas [4]. High-grade serous ovarian cancer represents the most common histologic type of EOC. It typically presents at advanced stage (III–IV) and, despite the initial response to surgical debulking and first-line therapy with carboplatin and paclitaxel (with or without bevacizumab), most tumors eventually develop drug resistance, with a 5-year survival generally below 30% [5].

**Fig. 1:**
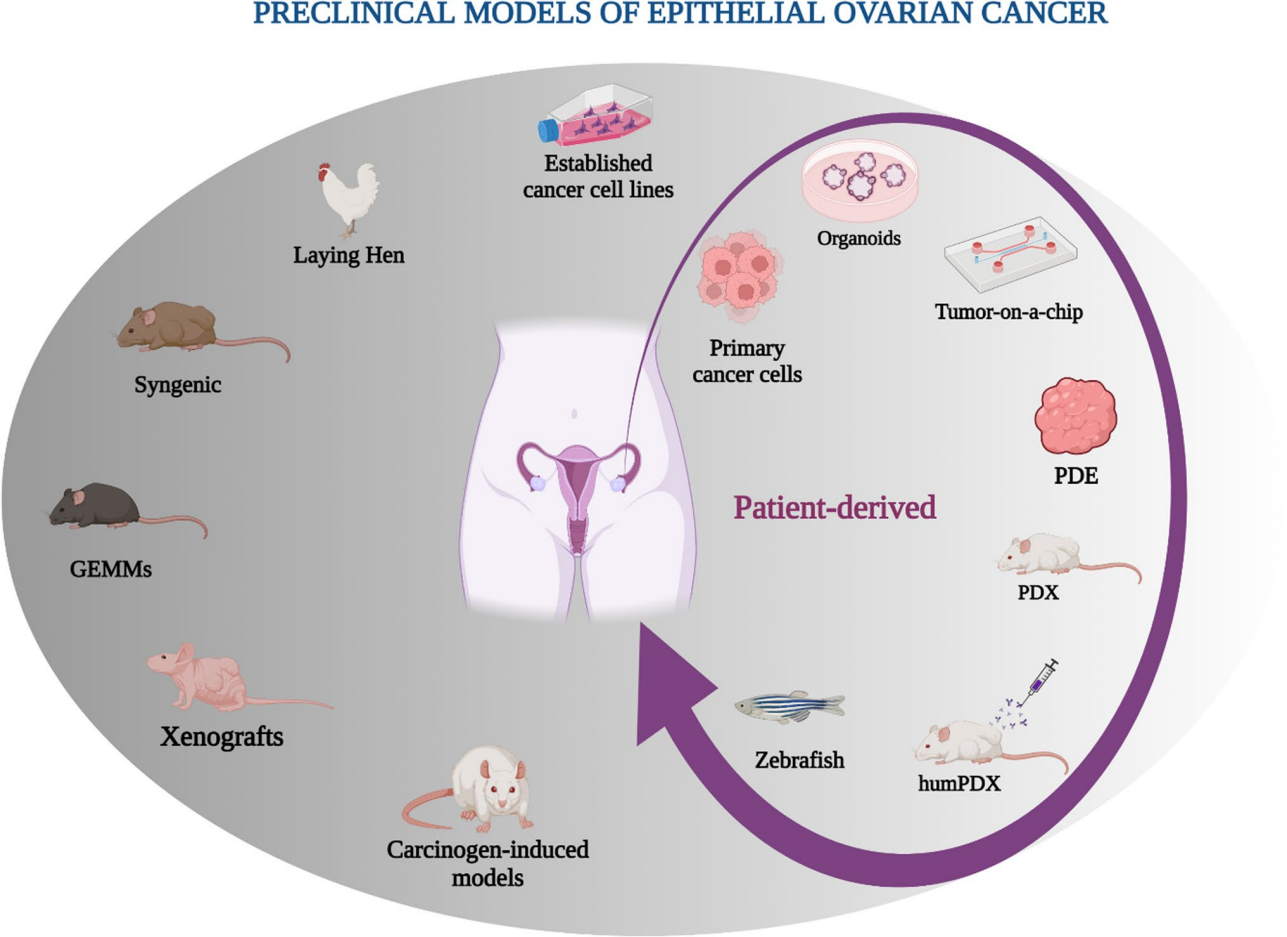
An overview of preclinical models of Epithelial Ovarian Cancer (EOC). *PDE* patient-derived explants, *PDX* patient-derived xenograft, *humPDX* humanized PDX, *GEMMs* genetically engineered mouse models. This figure was created with BioRender.com

Future high-quality translational research on EOC is therefore expected to focus on improving understanding of disease biology, identifying correlates of response and resistance to therapy and on providing new target cancer therapies, ultimately developing more effective ways to detect and treat this lethal disease. Proper selection of preclinical models and design of studies is mandatory for achieving such ambitious objectives, making preclinical data translatable to the clinic. This review is intend to offer a comprehensive overview of preclinical models available for the study of EOC (Fig. 1).

## In vitro and ex vivo models

### Established cancer-derived cell lines

Established cancer-derived cell lines have provided invaluable experimental tools for many decades to study cancer biology, identify correlates of response and resistance to existing therapy, and test the therapeutic efficacy of potential new treatments. Established cell lines are indeed relatively easy to manipulate, inexpensive to use and provide rapid experimental results. However, there have been questions about how relevant the research performed on these cell lines is, particularly with regard to misidentification and contamination of the cell line [a lists of cell lines that are known to be cross-contaminated or otherwise misidentified can be accessed here: https://iclac.org/databases/cross-contaminations/]. Besides, prolonged cell culture is likely to induce the occurrence of secondary genomic changes, including copy-number variations and transcriptomic drifts, or selection of some specific clones, elements that can bias the experimental results [6, 7]. Actually, although it appears that at the genomic level driver mutations are retained, literature data suggest that cancer cell lines resemble each other more than their original clinical samples, this limiting their usefulness and impacting the final overview [6, 7]. Because of these reasons, responses of some cell lines to drug, either in in vitro or in vivo preclinical models, were not recapitulated in many clinical trials, limiting the use of cell lines as a preclinical model [8]. In this context, the requirements for cell line authentication by short tandem repeat (STR) profiling have become stringent. CLASTR, the Cellosaurus STR similarity search tool (https://web.expasy.org/cellosaurus-str-search/) [9] enables users to compare STR profiles with those available in the Cellosaurus cell line knowledge resource, thus aiming researchers in the process of cell line authentication. Finally, contamination by mycoplasma and other microorganism has to be excluded before using.

In the context of EOC, the now well-recognized genomic heterogeneity of this disease adds further complexity to this already complex picture. Accordingly, in the last decade, several studies have been carried out to evaluate the suitability of the different available cell lines as representative models for the distinct EOC subtypes. Worldwide, there are about 100 ovarian cancer cell lines and near 70 of these are available at different cancer cell line bank, including ATCC (American Type Culture Collection, USA, https://www.atcc.org/); ECACC (European Collection of Cell Cultures, UK, https://www.phe-culturecollections.org.uk/collections/ ecacc.aspx); DSMZ (Deutschen Sammlung von Mikroorganismen und Zellkulfuren, Germany, https://www.dsmz.de/); RIKEN (RIKEN Bioresource Center CELL BANK, Japan, https://cell.brc.riken.jp/en/); JCRB (Japanese Collection of Research Bioresources Cell Bank, Japan; http://cellbank.nibio.go.jp/); CBA (CellBank Australia; http://www.cellbankaustralia.com/) [10, 11]. Only recently, however, some of the EOC cell lines from the Japanese collections have been made available from supplier located in Europe.

Noteworthy, a pivotal study by Domcke and colleagues [12] demonstrated that the most frequently used EOC cell lines seem for the most part badly suited for investigating HGSOC, the most prevalent EOC subtype, whereas the cell lines that more closely resemble these tumors are rarely used in laboratories. Using available molecular profiles of cell lines from the Cancer Cell Line Encyclopedia (CCLE) (47 cell lines examined) and comparing tumour sample data obtained from TCGA, the authors proved that significant differences exist in the molecular pattern between commonly used cell lines and HGSOC samples. To best differentiate between HGSOC and other EOC subtypes, they chose to evaluate both alterations typical of HGSOC (such as mutations in *TP53* and *BRCA1/2*, and amplifications in other genes including *CCNE1*, *MYC*, *PIK3CA*, and *KRAS*) and also mutations in a subset of genes classically altered in other EOC subtypes (e.g., *KRAS* and *BRAF*). Results obtained showed that the most recurrently mutated genes in HGSOC were also mutated in a significant fraction of cell lines; *TP53* was found mutated in 62% of cell lines, and *BRCA1* and *BRCA2* in 6% and 9%, respectively. A similar degree of copy-number alteration (CNA) was also demonstrated. However, among the commonly used models for HGSOC subtype, three cell lines, namely IGROV-1, SKOV-3, and A2780, had little profile similarity to the tumors, the latter also showing intact *TP53* [12]. Conversely, other cells, including less commonly used lines, such as COV362, COV318, and OV-90, were reported to be likely/possibly high-grade serous [12]. Other studies were published thereafter with similar results. Beaufort and colleagues [10], through a comprehensive profiling of 39 commercially available cell lines, assigned half of them (20) as non-serous type, 14 as high-grade serous, and five as serous-type/low-grade serous. Different approaches for the classification of EOC cell lines have been proposed, including the use of predictive clinical algorithms, as Calculator for Ovarian Subtype Prediction (COSP) [13] and of a transcriptional classifier developed by trialing machine learning algorithms [14]. Notably, in line with observations by Domcke and colleagues [12], results from these latter studies questioned the use of SKOV-3, A2780, and IGROV-1 as models of HGSOC, classifying these lines as derived from endometrioid/clear-cell EOC, although some uncertainties still exist. Other cell lines, including OVCAR-3, OVCAR-4, CAOV-3, KURAMOCHI, and OVSAHO, have been consistently classified as HGSOC [10, 12–14]. Table S1 shows the classification of a panel of EOC cell lines, according to the above-mentioned literature data.

With regard to HGSOCs, besides *TP53* mutations occurring in about 96% of cases, *BRCA1/2* germline and somatic mutations are detected in about 20–25% of patients [15, 16] and therefore, cell lines with *BRCA1/2* mutations may be of particular relevance for some experimental studies. Mutations in BRCA1 have been described in the cell lines COV362, JHOS2, and UWB1.289 [12] (https://depmap.org/portal/ccle/; https://cellmodelpassports.sanger.ac.uk/); likewise, *BRCA2* mutations have been found in KURAMOCHI and PEO1 cell lines.

However, it is worthy to note that cells with BRCA deficiency do not survive well in standard cell culture conditions and this can induce a selective pressure for reversion of the original mutation. This has been demonstrated in PEO1 cells by Stronach and colleague [17] who identified a *BRCA2* reversion mutation (5192A > T;5193C > G [Y1655L] in unselected stock of PEO1, in line with the previous findings from Sakai and colleagues [18], who reported that the same mutation emerged following selection with cisplatin/PARP [Poly (ADP-ribose) polymerase] inhibitors. Notably, sequence verification of our stocks of PEO1 has also identified this BRCA2 reversion mutation [c.4964_4965delinsTG (coverage: 1549/1653X: VAF:94%) p.(Tyr1655Leu)] (unpublished data). Cells with this mutation are homologous recombination competent and possibly exist as a sub-dominant population within the original PEO1 line, a condition that can alter cell growth and sensitivity to drugs.

Normal controls of cancer tissues are also required for comparative studies; both ovarian surface epithelial (OSE) cells and fallopian tube epithelial (FTE) cells represent cells of origin of HGSOC, while LGSOC are thought to evolve in a stepwise fashion from the OSE [19, 20]. Primary culture of normal cells, both OSE and FTE, is challenging due to the limited proliferative potential and early senescence or spontaneous transformation, in small cases. Different methods for culturing primary normal cells have been developed to obtain valuable experimental tools for studying transformation [21, 22]. However, their use is limited to single short-term and small-scale in vitro experimentation and the immortalization is necessary [22, 23]. The common immortalization process by induction of HPV16 E6/E7 and SV40 large T antigen expression, or in alternative hTERT, extends the lifespan of these cells, but is not sufficient for cell transformation. Importantly, immortalized OSE and FTE cells may be highly useful as control cells for OC research, especially for transformation assays in which they are transduced with different genetic alterations to reproduce the carcinogenesis process. Overall, studies evaluating disease biology and neoplastic progression may take advantage of these experimental tools, including the most appropriate model according to the specific histotype under investigation.

### Primary ovarian cancer cell lines

The establishment of primary, patient-derived, tumour cells provides a very important experimental system for better understanding EOC biology and mechanisms of therapy resistance, and for improving development of new drugs for personalized treatment. Indeed, despite having a limited lifespan and being slow-growing, primary EOC cells are valuable, because they preserve patient-cell features and can be associated with the clinicopathological data. On the other hand, immortalized cell lines, grown through serial passages, with genotypic and phenotypic changes, are less representative of the original tumour and their use might not be translationally relevant.

The isolation of primary EOC cells can been achieved using solid tumour specimens or patients' ascites with different methods [21]. To obtain single-cell suspensions from surgical biopsy, dissociation is the first step and three main dissociation techniques exist, based on chemical, mechanical or enzymatic processes [24]. Tissue dissociation is a critical issue, since, due to an excessive dissociation process, epithelial cells can lose their morphology. Besides, exerting excessive chemical or mechanical pressure on cells is a stressful factor that may significantly change the expression levels of genes. A major problem regarding the isolation of EOC cells directly from solid tumours is the presence of multiple cell types, namely erythrocytes and fibroblasts, and the composition of primary cultures varies, as function of tissue of origin. These contaminating cells may be present at initial plating, but most of them are removed using appropriate enzymatic digestion and at the media change, leaving the adherent EOC cells [21, 25]. Clearly, it is particularly important to determine whether the cells recovered are representative of tumour and the percentage of cell contamination. EOC mainly typically express cytokeratins in their cytoplasm; therefore, cytokeratin immunostaining is used for the identification of epithelial cancer cells.

Ascites fluid can also be a valuable source of tumour cells, easily accessible following paracentesis from the patient. Isolation and culturing of primary cancer cells from ascites have become common, and different approaches have been developed [26]. The isolation occurs without mechanical or enzymatic digestion [21] and, under non-adherent conditions, cells grow in aggregates, preserving their molecular and phenotypic profiles. Notably, in physiological conditions, ascitic cells can appear as multicellular aggregates (MCAs) or single cells [21, 27], although the development of aggregates facilitates tumour cell survival, protecting them from anoikis, and contributing to secondary lesion formation in peritoneal organs [28]. Latifi and colleagues [27] separated from ascites of HGSOC patients, two different populations: epithelial tumorigenic (non-adherent cells) with selective CSC-like markers and mesenchymal non-tumorigenic populations (tumour adherent), demonstrating that spheroids with cancer stem cell characteristics show a more aggressive metastatic and chemoresistant phenotype.

Culture of primary cancer cells is critical, due the slow growth capacity of cancer cells, the limited overall lifespan, as well as the non-tumour cells contamination of the culture, factors inducing a lack of reproducibility. Indeed, cancer cells frequently lose growth potential after some passages and go into crisis, suggesting that replicative senescence might be a crucial step in becoming a cell line under culture conditions. Recent findings have shown that primary EOC cells become growth-arrested after approximately five population doublings [29, 30], being their proliferative capacity homogenous across different histotypes [29]. Senescent cells, exhibiting biochemical and molecular signatures of senescence, were shown to be growth-arrested in the G1 phase of the cell cycle, the stage where the majority of normal cells undergo replicative senescence. Notably, the size of the senescent EOC cells fraction was smaller (below 10%) compared with the other cancers [29, 31]. It has been suggested that these cells appear in culture conditions because of their direct transfer from the tumour mass and also as a consequence of their high vulnerability to environmental insult (culture shock) [29]. According to Pakula and colleagues [29], primary EOC cells undergo spontaneous senescence in a mosaic, telomere-dependent and telomere-independent manner. Finally, senescence in primary EOC obtained from ascites also occurs between the 2nd and 8th passages [32]. Therefore, to ensure a healthy and proliferative starting material, experiments on primary EOC cells should be performed within passages 2–4.

The medium for tumour primary cell cultures plays an important role for growing and maintaining them, without causing any genetic drift. Various nutrients and growth factors (epidermal growth factor, insulin, hydrocortisone, beta-estradiol, and progesterone) may be added to the medium even if their addition can alter the growth and epithelial morphology of EOC cells [33, 34]. Ince and colleagues [35] successfully established 25 novel cell lines from primary OC with significant high rate using culture media and conditions optimized to each histological subtype. Notably, these established cells retained the genomic landscape, histopathology, and molecular features of the original tumours. Furthermore, the drug response of these cell lines correlated with distinct groups of primary tumours with different outcomes [35].

Overall, primary EOC cell lines by preservation of cell phenotypes, stemness, and heterogeneity of cancer subpopulations offer advantages not always attainable by established cell lines, thus representing a significantly improved platform to study human tumour pathophysiology and response to therapy.

### Two- and three-dimensional cell culture models

The traditional 2D cell culture method is a well-known, inexpensive and relatively easy system to generate and maintain cell lines and to evaluate response to drug treatment. In 2D cell culture conditions, cells grow and expand two-dimensionally on the surface of cell culture dishes, thus taking a flat and elongated shape, being uniformly exposed to nutrients, growth factors, and test agents [36]. Although 2D cell culture is generally accepted and still used because of its simplicity and low cost, there are limitations associated with it. In this culture method, cell–cell and cell–extracellular environment interactions are not represented, gene and protein expression levels are often different compared to in vivo models, and analysis of response to cytotoxic drug treatment may overestimate drug efficacy. Indeed, many properties of organs and tumours, including tissue architecture, cell–cell and cell–matrix interactions, mechano-physical properties, and gene expression networks are not, or only partially, represented under 2D culture conditions. These limitations of the 2D systems have prompted research on alternative models, better able to mimic a natural tumour mass, such as three-dimensional (3D) culture systems. Therefore, switching from 2 to 3D cultures has been moved by the need to create cellular models that could better recapitulate the complexities of tumour biology. Besides, 3D models have been recognized as proper tools for drug discovery and screening.

Three-dimensional cell cultures are classified as scaffold-based or non-scaffold-based techniques [37]. Scaffold-based culture technologies provide physical support on which cells can aggregate, proliferate, and migrate; scaffolds can be of biological origin or synthetic, to mimic key properties of the extracellular matrix (ECM) [37]. More recently, this scaffold-based approach has been used by 3D bioprinting technique to create more complex models with well-defined architecture, composition, and high reproducibility [38]. On the other hand, the scaffold-free-based 3D systems occurs through self-aggregation of cells, with development of multicellular aggregates, commonly known as spheroids [39]. Spheroids can be obtained from established cell lines or patient-derived tissue samples, although not all primary tumour cells or conventional cell lines are capable of forming spheroids [40]. Heredia-Soto and colleagues [41] obtained spheroids from 16 commonly used, commercially available OC cell lines, with three different patterns for 3D cell growth. Some cell lines adopted a loose aggregate conformation (i.e., A2780, A2780Cis, OVCAR-3, OAW28, PEA1, PEA2, PEO23, and TO14), others had a more compact aggregate and non-spherical structure (i.e., PEO1, PEO4, PEO6, and PEO14), and a third group of tight spheroids had very well-defined perimeters (i.e., PEO16, OV56, SKOV-3, and 59 M). Overall, it has been reported that ovarian spheroids show morphological resemblance to multicellular aggregates in cancerous ascites [42]. Analysis of spheroid versus monolayer ovarian cancer cells has demonstrated differences in the expression of several biomarkers relevant to disease, which could alter the tumorigenic properties of the cells [43]. Overall, these findings support the hypothesis that ovarian cells in 3D culture are physiologically different from their 2D monolayer, indicating 3D growth more informative in studying the properties of EOC cell lines. Besides, these aggregates displayed a higher chemoresistance after paclitaxel and cisplatin treatment, when compared to 2D condition, mimicking the in vivo response [43]. Indeed, as a peritoneal metastasis, the access of chemotherapy agents to internal cells can be inhibited in the un-vascularized 3D spheroids due to their structure characterized by a metabolite density gradient, this possibly representing a mechanism of resistance in EOC [44, 45].

To mimic the cancer niche and the interactions between the tumour and its microenvironment, 3D co-culture models have been also established from cancer cell lines or primary cells, in combination with stromal cells as fibroblasts, endothelial, or immune cells. Recently, Long and colleagues [46], by co-culturing OC spheroids and tumour-associated macrophages (TAM) have shown that the interaction with TAM promotes the progression of OC. These findings support the translational relevance of such experimental models. Indeed, it is known that the most abundant population of tumour-infiltrating immune cells in EOC are TAMs, which have been demonstrated to play a critical role in development of tumour progression and chemoresistance [47–49].

### Organoids

Organoids can be defined as 3D structures derived from stem cells of various organs and tissues. They are derived either from adult stem cells (ASC) or from pluripotent embryonic stem cells (ESC) and their synthetically induced counterparts, i.e., induced pluripotent stem cells (iPSC) [50]. This important feature denotes the major difference between spheroids and organoids; organoids originated from stem cells, whereas spheroids not.

Unlike other type of cancers, only a limited number of studies are available for patients-derived organoid (PDO) cultures from EOC. To establish PDOs from resected OC biopsies, the primary tumour tissue is initially digested by mechanical and enzymatic digestion followed by embedding cells into a specific matrix (such as Matrigel) and culturing medium, supplemented with a cocktail of growth factors and hormones for long-term maintenance. Different experimental protocols have been set up to obtain EOC organoids. A detailed description of methodological approaches is out of the scope of this review, but exhaustive information can be found in recent reviews [51, 52]. A critical aspect of organoid culture is certainly the definition of a growth factors cocktail, since differences in medium components are important to enhance the efficiency of kick-starting organoid cultures from individual patients. However, components of culture medium differ very much among different researchers. Indeed, according to Kopper and colleagues [53], a Wnt-conditioned medium may be essential on some cell lines, while detrimental in other cases, irrespective of the histotype considered. On the other hand, Hoffmann et al. [54] suggested that the Wnt pathway’s inhibition could promote the growth of HGSOC organoids. Beside, other growth factors and signaling molecules, including epidermal growth factor (EGF), noggin, R-spondin1, nicotinamide, and the Rho kinase inhibitor, Y-27632 are commonly used in EOC organoid cultures [51, 52]. On the whole, it appears from available literature data that experimental conditions for EOC organoids culture should be standardized by experts.

First published data illustrated organoid cultures obtained from few EOC patients [55, 56] and/or short-term HGSOC organoids [57]. However, in a recent article by Kopper and colleagues [53], a main development in EOC organoids was published. PDOs were obtained from non-malignant BOTs, as well as MOC, CCC, EnOC, LGSOC, and HGSOC with an overall success rate of 65%; notably, even after extended passaging, PDOs have been shown to morphologically and molecularly match the parent tumors from which they were derived [53]. In addition, organoids recapitulated EOC hallmarks, such as CNV, recurrent mutations, and tumour heterogeneity. Authors also obtained OSE- and FTE-derived organoids, and they used gene manipulation technologies (CRISPR/Cas9 Gene Editing) to assess the potential of these experimental models to study early HGSOC development. Likewise, Maru and colleagues [58] documented faithful duplication of histological features and tumour heterogeneity within PDOs derived from various subtypes, including HGSOC, MOC, and EnOC. Later publications have corroborated the potential of PDOs to be employed for drug screening, as well as for studying OC biology and mechanism of drug resistance [54, 59–63].

Overall, research findings support the use of PDOs as an attractive platform for modeling EOC, drug–response prediction/patient selection and for high-throughput drug screening. A longitudinal observational phase II, single-center, single arm study (NCT04555473) is now ongoing in our Institution to evaluate the reliability of HGSOC organoids obtained from primary debulking surgery (PDS) + adjuvant chemotherapy and neoadjuvant chemotherapy + interval debulking surgery (NACT + IDS) cases, as model for the patients' response to treatments. We also aim to study the genomic and phenotypic evolution of tumour cells in HGSOC organoids from PDS + adjuvant chemotherapy and NACT + IDS patients undergoing relapse. Other clinical trials are also ongoing worldwide to evaluate the role of PDOs in predicting the clinical efficacy of anticancer drugs in OC (NCT02732860, NCT04279509, NCT04768270, NCT05175326, and NCT05290961). Results from these trials will help defining the consistency of these models as avatars for human disease and their use in coclinical studies. Currently, collections of EOC PDO and matching healthy organoids are generated and biobanked to be employed for screening of new drugs or new drug combination.

However, the model has some important drawbacks: the process is time-consuming, with a high variability in the success rate and time of establishment, partly because of biopsy quality or size. Indeed, organoid development efficiency is strongly dependent on the viability of the cells after dissociation, in turn linked to primary patient characteristics, including tumour histotype, grade, and cell composition of the clinical specimen. Low tumour purity can also influence genomic correlation between PDO and tissue [53]. Mostly important, organoids are devoid of the native microenvironment, with a lack of vasculature, tumour stroma and immune cells, factors playing a critical role in translational cancer research. Therefore, drugs targeting tumour microenvironment, including anti-angiogenic and immunotherapeutic agents, cannot be tested in tumour-derived organoids [64]. Besides, it should be kept in mind that tumour microenvironment (TME) may alter drug response, determining discrepancies between drug sensitivity in vitro and in vivo [65]. The development of co-culture conditions of organoids with immune cells or other cells may overcome these limitations. Notably, Wan and colleagues [66] have recently generated short-term co-cultures containing tumour organoids and the full complement of intratumoral immune cells from 12 solid tumors of HGSOC patients to test a unique bispecific anti-PD-1/PD-L1 antibody compared with monospecific anti-PD-1 or anti-PDL1 controls.

### Tumour-on-a-chip

Recently, tumour-on-a-chip systems have emerged as a powerful tool for studying tumour biology, metastatic pathways, and drug screening. These systems consist in a microfluidic device, obtained with advanced microfabrication techniques, where different cell types are seeded within separate chambers to recreate the dynamics found in the TME [67]. Typically, microfluidic models are obtained by seeding and culturing cells in 3D scaffolds in a small chamber, under perfusion of culture medium. The microfluidic perfusion permits an accurate control of microenvironment, and manipulation of physical and biological parameters. Importantly, the latest microfabrication techniques allow the integration with different component of TME as stroma and immune system cells. Specifically, Saha and colleagues [68] have developed an OvCa-chip in which A2780 cells or primary cells obtained from HGSOC patients were co-cultured with endothelial cells in two overlaid microfluidic chambers separated by matrix-coated porous membrane. To mimic platelet extravasation dynamics, the vascular lumen of the device was perfused with platelets, suggesting an active role of OC cells in this mechanism [68]. More recently, the same authors developed an ovarian tumour microenvironment chip (OTME-Chip) that, in addition to the tumours interfacing platelet-perfused vascular endothelial tissue, also incorporates an adjacent well-defined collagen hydrogel-based ECM microenvironment. This platform has been also integrated with gene editing and next-generation RNA sequencing (RNA-seq) tools to study vascular and hematological targets in OC [69]. Likewise, Surendran and colleagues [70] have developed a 3D tumour/neutrophils-on-a-chip device in which EOC spheroids produce an in vivo-like immune response associated with neutrophil chemotaxis.

Overall, although tumour-on-a-chip allows to achieve valuable results for the EOC studies, some critical issues need to take into account regarding its applicability, including the complexity of design and use, the limited biomaterials choice, and the weak standardization using commercially available cell lines.

### Ex vivo models

Ex vivo models are mostly represented by patient-derived explant (PDE) in which fresh surgically resected tumour can be cultured ex vivo (entirely or in slices, with or without a cellular matrix) for a period of time. These models potentially maintain the spatial conformation of the tissue, heterogeneity, and tumour grade, and therefore, they can successfully be used for studying cancer biology and developing personalized treatment.

Over the time, different ex vivo culture methods have been developed by different research groups, with different tumors requiring different culture conditions. Briefly, explants may be maintained as fragments or processed for generation of tissue slices of around 300 μm and subsequently cultured as free-floating culture or using grid/pore membrane supports, or gelatin sponge supports [71]. Use of tissue slices facilitates drug diffusion, but may result in the loss of tissue architecture. Slicing methods reported for EOC tissues include manual dissection with a scalpel [72] and the mechanized sectioning systems Krumdieck [73–75] and McIlwain Tissue Chopper [76]. Manual slicing is becoming less common due to the lack of uniformity in explant thickness, while the Krumdieck tissue slicer is the most used in OC research, being considered adequate for the purpose of developing precision tissue slices for subsequent culture. Another instrument described for fresh tissue sectioning is the vibratome that, compared with other instruments available, has been suggested to better preserve the integrity of delicate samples, thus ensuring a higher number of viable cells on the section surface [77]. However, there appears to be little information on the use of this latter method in precision slicing of EOC, and therefore, further studies are needed to define the golden standard approach. For incubation conditions most studies use 37 °C, 5% CO_2_ and 21% oxygen [71, 78]. Further details on culture conditions can be found in a focused review by Templeton et al. [78]. Tumor explants cultures can also be combined with adjacent healthy tissue or cell culture, as for example lymphocytes from peripheral blood mononuclear cells (PBMC).

Endpoint analysis for studies assessing drug response includes either enzymatic digestion followed by evaluation of cell viability or cytotoxicity using the MTT (3-(6)-2,5-diphenyltetrazolium bromide) assay [as in the case of Histoculture drug response assay (HDRA) assay [79]] or the PDE can be left intact and processed for spatial biomarker analysis [78]. If the PDE is homogenized, protein and nucleic acids can be extracted and omics data generated.

Overall, limited literature data are available on patient-derived explants of EOC. Indeed, besides studies reported above, showing that the ex vivo explant assay is a robust and cost-effective model to assess chemosensitivity and the effect of novel therapeutics in EOC [71–76], a few more investigations have been carried out applying the HDRA assay to the prediction of drug response in EOC [80, 81]. Specifically, Nakada and colleagues [80], using a modified HDRA in a total of 164 patients, reported a high evaluability rate and a strong correlation with the clinical response. Similar results were reported later by Jung and colleagues [81] who, in a prospective clinical trial, found a significant association between the in vitro HDRA chemosensitivity to carboplatin and paclitaxel, and the PFS of patients with advanced EOC. Recently, however, the applicability of HDRA to predict platinum sensitivity and prognosis in EOC has been questioned by Lee and colleagues [82].

Interestingly, to improve the longevity and preserves the histopathological features of EOC explants, Abreu and colleagues [83] have recently developed a long-term agitation-based EOC-PDE culture platform that retains the tumour microenvironment and patient-specific features. According to author’s conclusion, this experimental model may allow to explore disease mechanisms, to test new drugs, and to elucidate drug response and resistance mechanisms, due to the feasibility of cyclic drug treatments.

The main drawbacks of these ex vivo methods are the limited accessibility of fresh tissues, the lack of reproducibility, due to the natural heterogeneity of donor tissues (or possibly due to the small amount of tissue that could not reflect the cancer heterogeneity), and the limited cell viability (of few days), as a possible consequence of limitation in the diffusion of nutrients and oxygen.

## In vivo models

In EOC studies, three species are most commonly used, i.e., mouse, rat, and lying hen. The laying hen is distinctive in being the only model that allows observations of early events in disease progression, and indeed, it is suited for chemoprevention studies. On the other hand, rodent models represent the gold standard for tumour growth and tumour response to drug compounds, although limitations exist including ethical controversy, the species-specific differences between animals and humans, low‐throughput drug optimization, and animal expenses. The most widely used rodent models in EOC research include xenograft, syngeneic, and genetically engineered models [84, 85].

For xenografts and/or syngeneic models, three important factors need to be taken into account in the context of EOC: (i) tumour cell source, i.e., established or primary cancer cell line, or surgical resections; (ii) location of transplanted tumour cells (orthotopic *vs* heterotopic); and (iii) immune status of the host (mouse immune system, immunocompromised or human immune system). The two major methods of engraftment are subcutaneous (SC) and intraperitoneal (IP) injections. After subcutaneous engraftment of cancerous cells or tissues, tumour formation is confined to the place of implantation and grows within weeks, showing histology similar to the original tumour. It can be readily quantified with calipers, rendering it a suitable model for studies of drug response. IP injection allows obtaining a disseminated cancer model, mimicking metastatic behavior of EOC. Cancer foci are quickly formed within the peritoneum, on the liver and spleen surface, similar to the advanced stages of human EOC. Only a suspension of established or patient-derived cancer cell lines can be injected via IP. In orthotopic mouse models, tumour cells are transplanted in the anatomical location from which they were originally derived, as ovarian bursae (IB, intrabursal) [85]. IB injections are technically challenging and require skill and experience, possibly resulting in low implantation rate and tumour size variability among mice. Besides, the anatomical difference between mouse and human ovaries (the bursal membrane is a unique feature in mice) may affect the ability of cancer cells to leave the primary site of injection in IB models. Importantly, the orthotropic model has similar TME as the original tumour and therefore more closely resemble the tumorigenesis in patients, in terms of vasculature, gene expression, response to chemotherapy, and metastatic biology [86]. Orthotopic implantation or IP injection of EOC cells are thus more clinically relevant, but more complex when analyzing changes in tumour growth. This can be monitored by different methodology including magnetic resonance imaging (MRI), ultrasound (US), and bioluminescence imaging. MRI and US are used to determine volumes and internal structure of the tumours, monitoring the tumour growth and metastasis [87]. However, these approaches in preclinical studies are extremely expensive and time-consuming, especially with a large number of mice. Using EOC cell lines stably transfected with a luciferase-expressing gene, the tumour burden, including metastasis, can be analyzed by measuring bioluminescence emission using In Vivo Imaging System (IVIS) [88]. This approach is much simpler and economical, but cannot be applied to engraftment with tumour tissues. Overall, the inherent difficulties in monitoring IP or IB disease formation and progression (unless specific equipment for the in vivo imaging system are available) may increase the risk of the animals' distress [89]. Consequently, the choice of appropriate humane endpoints is more difficult in these models, an issue that can be questioned by the Ethical Committees for animal experimentation. In conclusion, taking into accounts all these aspects, the use of SC xenografts may be considered at the early stages of in vivo preclinical evaluation of a new drug. If encouraging results are achieved, they have to be confirmed in mid- to late-stage research in more clinically relevant models.

Features relative to tumour cell source and immune status of the host are described below.

### Carcinogen-induced tumour models

Numerous studies have reported the use of 7,12‑dimethylbenz[a]anthracene (DMBA) to induce ovarian tumors in rodents, mainly in rats, by exposing ovaries to the carcinogen either by introduction of a DMBA-saturated suture/gauze under the ovarian surface, or by injection of DMBA directly into the ovary [90–95]. Importantly, however, the animal strain used, the age of the animals, or the use of concomitant hormone treatment was shown to significantly affect the rate of tumour formation as well as the histologic types of experimental ovarian tumours, with a variable histological distribution, including both epithelial and sex-cord stromal tumours. Thus, although these models have the advantages of encompassing all stages of the neoplasia, the lack of standardization has actually limited their use.

### Syngeneic models

In syngeneic models, also known as allograft models, tumour cells (or tumour tissues) derived from a particular inbred strain are engrafted into hosts of the same strain. These models allow studying the interaction between the tumour and the immune system, as well as the effects of immunotherapies on tumour and surrounding immune cells.

Currently used models for EOC include the commonly used ID8 murine model [96] together with genetically modified versions of these cells [97–101]. The syngeneic mouse model permits EOC initiation directly from mouse OSE (MOSE). MOSE cells scraped from the mouse bursa were passaged in culture on plastic until phenotypic changes occurred, such as the loss of cell contact inhibition, which resulted in cellular mounds and changes in cell morphology [96]. Late passage MOSE (clone ID8) cells injected IP into syngeneic C57BL/6 mice gave rise to peritoneal tumours with ascites, within about 90 days; tumour formation following SC injection occurred in ~ 4 months [96]. Later, Greenaway and colleagues [102] demonstrated that orthotopic grafting of ID8 cells into C57BL/6 mice could induce, between 80 and 90 days post-injection, the formation of epithelial ovarian tumours and secondary lesions throughout the peritoneal cavity, with cytological and architectural features resembling serous carcinoma; extensive abdominal ascites was recorded, as well.

Nonetheless, genomic analysis has shown that ID8 tumours do not carry the common mutations and somatic copy-number alterations observed in human HGSOCs, such as those occurring in *Trp53*, *Brca1*, and *Brca2* [99]. Using CRISPR/Cas9 gene editing, Walton and colleagues generated sublines of ID8 that recapitulate critical mutations in human HGSOC, as single (*Trp53*–/–) or double mutants, with deletions in *Brca1*, *Brca2*, *Pten* and *Nf1* in addition to loss *Trp53*, as well as triple mutants lacking *Trp53*, *Brca2* and *Pten* [99, 100]. Collectively, their results indicate that these cell lines can represent powerful models to clarify HGSOC biology and chemotherapy resistance, demonstrating that tumours derived from differing mutations respond differently to treatments and result in alterations in immune cell infiltration into the tumour microenvironment. More recently, Iyer and colleagues [103] engineered a panel of murine fallopian tube epithelial cells bearing mutations typical of HGSOC and capable of forming tumours in syngeneic immunocompetent host. Interestingly, the models were set up to reproduce molecular pathway occurring in homologous recombination (HR)-deficient or HR-proficient patient population and their clinical relevance was further corroborated by their responsiveness to both DNA-damaging agents and PARP inhibitors. These experimental systems could be particularly important to address the clinical unmet need of alternative therapeutic options for patients with HR-proficient HGSOC. Besides, they might identify predictive biomarkers to improve women response rates under treatment, particularly in the field of immunotherapy.

### Xenografts of established ovarian cancer cell lines

Xenograft models have been largely used in EOC research and are still very important for preclinical drug screening. These models require use of immunodeficient mice strains that show a decreased immunological response. Several strains are available: the athymic nude mice lacking T lymphocytes (Foxn1 Nu/Nu, with spontaneous deletion in forkhead box N1 gene); the severe combined immunodeficiency (SCID), depleted of functional B and T lymphocytes; the non-obese diabetic (NOD)/SCID and the NOD/SCID/IL2Rγ null mice (NSG) deficient in mature lymphocytes and NK cells [104]. Established cell lines have been generally used for xenotransplantation experiments, showing variable ability to grow in nude mice when implanted SC, IP, or IB. The rate of engraftment of human OC cell line can be improved by mixing them with Matrigel [105].

Overall, available data suggest that some cell lines are tumorigenic in both SC and IP locations, while others exhibit a strong propensity to grow in one site only, this implying that the TME can reprogram different signaling pathways for tumour proliferation. Shaw and colleagues [106] evaluated tumour formation after IP injection of 11 EOC cell lines (HEY, OVCA429, OVCA433, OCC1, OVCAR-3, SKOV-3, A2780-s, A2780-cp, OV2008, C13* and ES-2) in nude mice to characterize their growth patterns and disease histology. ES-2, OCC1, A2780-cp, and HEY were the most aggressive cell lines (median survival time < 30 days), while A2780-s, OVCA429, OV2008, and SKOV-3 cells were less aggressive (median survival time 2–3 months). Conversely, C13*, OVCA433, and OVCAR-3 cells failed to form IP tumours within 3 months. Histologically, A2780-s, A2780-cp, ES-2, HEY, and OCC1 were defined as undifferentiated carcinoma; OVCA429 and SKOV-3, as CCC; and OV-2008 and C13* as EnOC with foci of squamous differentiation [106]. Besides, comparison of tumour characteristic between IP and IB dosing showed that for both OVCA429 and ES-2, the site of injection did not affect the tumour histology, while the tumour take rate was negatively affected for OVCA429 cells [106]. Later, Mitra and colleagues [107] compared growth characteristics of IP and SC injection of different EOC cell lines (CAOV-3, COV362, KURAMOCHI, OVCAR-3, OVCAR-4, OVCAR-5, OVCAR-8, OVSAHO, OVKATE, SNU119, and UWB1.289) in female athymic nude mice. Each cell line displayed different growth characteristics in vivo. OVCAR-3 cells formed rapidly IP tumours with HGSOC histology, while OVKATE and COV362 formed only tumours by SC injection. Only OVCAR-8 formed ascites. Three cell lines (KURAMOCHI, SNU119, and UWB1.289) were non-tumorigenic. Likewise, also Hernandez and colleagues [108] evaluated in vivo tumorigenicity of a panel of EOC cells after SC, IP, or IB injection. They demonstrated that A2780, OVCAR-5, OVCAR-8, IGROV-1, SKOV-3, CAOV-4, PEO1, and MDAH-2774 were medium/highly tumorigenic via SC injection; with the exception of IGROV-1 and PEO1, the same cell lines also showed a medium/high ability to form tumours when injected IP; authors also reported that only OV-90, OVCAR-8, and CAOV-4 were highly tumorigenic in IB location. Notably, they showed that cell lines showing preference for IP growth had gene expression patterns more similar to primary tumours, although, histologically, the IP tumours appeared as undifferentiated carcinoma, without clear morphology of any human histologic subtype [108].

In our experience, PEO1 or COV318 cells did not form tumours in athymic nude mice injected either via IP or SC (unpublished data); on the other hand, A2780, OVCAR-3, SKOV-3, and HEY cells were tumorigenic after SC and IP injections [47, 109–111].

Established EOC cell lines in vivo have some advantages including fast tumour growth and intra- and inter-laboratory reproducibility. However, even if these models are particularly useful for drug screening, the immunodeficiency state does not allow to evaluate the contributions of the immune factors to tumour development. Besides, it is important to confirm cancer cell line identity, before injection into mice. Likewise, the histological subtype of tumour grown in mice needs to be evaluated by immunohistochemical and, possibly, mutational analysis.

### Patient-derived xenografts (PDX)

Patient-derived xenografts (PDXs) are generated by direct SC, IP, or orthotopic engraftment of clinical samples into immunodeficient mice; after the tumour reaches a critical size, it can be excised and implanted into subsequent mice. Surgically resected tumours, patient-derived cells, or samples from ascites can be used to produce ovarian cancer PDXs. There are advantages and disadvantages in utilizing either tumour fragments or single-cell suspensions. Indeed, tumour fragments maintain cell–cell interactions and the architecture of the original tumour, mimicking its microenvironment; on the other hand, the cell isolation procedures improve cell viability and engraftment success, although, during cell passaging, the population can enrich for subclones. Under the different experimental conditions, a wide variation occurs in the percentages of engraftment and time to develop tumours. With regard to the IB and IP route, available data reveal high engraftment rate (> 70%), particularly when SCID or NOD-SCID-IL2γR mice are used [112–115]. Weroha and colleagues [114] described the first large bank of EOC PDX, obtained after IP injection of tumour slurry in SCID mice. They reported an engraftment rate of about 70% (168/241), with microscopic fidelity and comparable genomic aberrations with the corresponding primary tumour. Notably, serous tumours displayed a higher PDX rate compared to other histotypes. They also demonstrated that responses to carboplatin and paclitaxel in vivo correlated well with the corresponding patient’s clinical response [114]. Similar findings were described by George and colleagues [115], developing over 40 PDX models using an orthotopic transplant approach in NSG mice with a 93% success rate (*n* = 37 of 40, time to take rate within 4 to 6 weeks of transplant) and 100% take rate for F1 and F2 generations. Interestingly, they also generated 14 orthotopic HGSOC PDX models with BRCA1/2 mutations (BRCAMUT) [115]. However, lower take rates were reported by other groups, although with some differences in experimental conditions. In detail, Ricci and colleagues [116] xenotransplanted in nude mice 138 tumour samples by SC, IP, or IB injection, achieving a 25% tumour take (34/138), regardless of the transplantation route. Median survival time was 1–4 months for IP transplanted xenografts, while time to reach 1 g was between 1 and 15 months for SC transplanted xenografts. Likewise, Liu and colleagues [117] generated PDX models in irradiated nude mice from IP injections of tumour cells isolated from the ascites or pleural fluid of patients: considering PDX models that successfully grew through at least three serial passages, they established 29 PDX, for a take rate of 31% (29/94). The latency time to development of clinically apparent disease from the time of initial implantation varied from 2 to 12 months. An even lower take rate after IP injection was reported by Dobbin and colleagues [118], who compared the take rate of different sites of transplantation, i.e., SC, IP, MFP (mammary fat pad), and SRC (subrenal capsule) in SCID mice. Specifically, they found a 22.2% take rate after IP injection, while obtaining higher values after MFP or SC xenotransplantation (63.64% and 85.3%, respectively). The lowest take rate was found for SRC implantation, i.e., 8.3% [118]. Conversely, Stewart and colleagues [119] reported that the injection of CD45-depleted serous OC cells (obtained from HGSOC patients) via IP, SRC, IB, or MFP route in NOD/SCID mice resulted in high tumour takes regardless of the transplantation route (> 70%). A limited number of studies have been carried out using the SRC route for tumour xenotransplantation. Besides those reported above [118, 119], also Lee and colleagues [120] reported a high rate of different histotype of EOC in SCID mice via the SRC route in a limited case series. Finally, Heo and colleagues [121] developed PDXs by SRC implantation of primary EOC tissues into female BALB/C-nude mice, with a rate of successful PDX engraftment of 48.8% (22/45 cases) and showed that patients whose tumors successfully engrafted in mice had inferior OS.

With regard to the SC route, Eoh and colleagues [122] successfully engrafted 49 out of 88 EOC specimens (53.4%) in NOG (NOD/Shi-scid/IL-2Rγnull) mice, suggesting that engraftment failure of chemotherapy-naïve tumors reflected low aggressiveness of the primary tumour. Likewise, Cybula and colleagues [123] associated the successful tumour engraftment rate to intrinsic features of the primary tumour reflecting its aggressiveness. Using NOD/SCID, NSG, or NRG (a strain very similar to NSG), they established a panel of HGSOC PDXs, by SC transplantation with an overall take rate of 77% (33/43) and a latency time from 4 to 10 months. They found no differences in tumour take rates between NSG and NRG mice, while slightly less in NOD/SCID mice. However, only 17 out of the 33 PDX engrafted could be further expanded through multiple rounds of serial transplantation [123].

It appears from the studies examined that a better engraftment rate can be achieved by implanting tumour tissues into SCID or NOD-SCID-IL2γR mice, rather than BALB/c nude mice. However, despite the more immune-compromised strains appear to have higher take rates, establishing PDX tumour models in NSG mice has presented some challenges and limitations. Indeed, several recent studies have revealed that OC PDX engrafted in NSG and NOG mice are susceptible to Epstein–Barr virus (EBV)-associated lymphomagenesis. Butler and colleagues [124] observed a lymphoma rate of about 11% in a panel of 117 EOC PDX. In line with these findings, preliminary data from our lab also show that a not negligible proportion of PDXs turn out to be human lymphocytic tumours (unpublished data). These lymphoproliferative lesions were consistently characterized by atypical growth kinetics with fast tumour growth generating soft, flat tumour masses. Evidence of lymphoproliferative tumours development after transplantation of OC PDX was also reported by Cybula and colleagues [123]. Notably however, in contrast to others, this study did not detect EBV-associated, but mouse lymphomas.

An important aspect to take into account is the molecular fidelity of PDX models to original human tumour. On the whole, available literature data comparing small numbers of PDX models and human tumors at the molecular level suggest that PDX models of EOC largely maintain molecular features of the original tumour [114–118]. Izumchenko and colleagues [125] also showed that the background mutation frequencies in EOC PDXs and primary TCGA tumors were highly comparable. However, some degree of genomic variation has been reported. Liu et al. [126] compared the gene expression profile of paired PDX and donor tumors, evidencing differences mainly related to the loss of human stroma in PDX tissues or reflecting changes required for a human tumour to adapt to a murine host [126]. Besides, despite an overall similarity, some degree of genetic evolution is expected in higher-passage PDX tumors and/or at the time of PDX initiation and adaptation to mouse host, due to clonal selection and/or clonal evolution [123, 127]. Chen and colleagues [128] also explored a relevant cohort of EOC patients and reported that PDX models generated in the study retained the protein expression, and genetic alteration patterns of the original tumors. Notably, despite the transcriptomic differences observed, the PDX models demonstrated a high degree of similarity with patients in terms of the chemotherapy response, indicating that non-driving differentially expressed genes (DEGs) not affected drug sensitivity.

It is also apparent from a number of the above-mentioned studies that a very good correlation exists between patient drug response and PDX response to the same drug [114–118, 125, 128]. In this respect, coclinical trials with PDX models have been initially proposed to form mouse-avatar models for conducting personalized treatment testing for the patient from whom the PDX was derived. However, the relatively long time required to complete in vivo studies has suggested that the possibility to use PDX response data to drive individual patient’s treatment is hardly achievable at this time. We searched on ClinicalTrials.gov for clinical trials on PDX/EOC (27 April 27 2022) and identified a total of three relevant studies, two completed from Mayo Clinic (NCT02283658 and NCT02657928) and one recruiting from Princess Margaret Cancer Centre Toronto (NCT02732860). For the two completed trials, the development of PDX avatars on tumors from participants was a tertiary objective. Both studies confirmed the feasibility of PDX from the majority of patients, but authors concluded that PDX coclinical trial attempting to use PDX response data to impact an individual patient’s treatment would be challenging, due to the evidence that time required to create a PDX commonly exceeded the patients’ time on study [129, 130]. For the ongoing trial (NCT02732860), the evaluation of the utility of PDX (comprehensively characterized by genomic and epigenetic analysis) as clinical predictors to direct the use of chemo- and targeted therapies in patients with different cancers, including HGSOC, is the primary outcome. Interestingly this trial also explores organoids to correlate between PDX and organoid drug sensitivities. Results of this study will provide further insights into critical aspects of the PDX models, as those related to their faithful representation of the original tumour and their genomic stability.

Different providers offer PDX models of EOC, although they are often quite expensive. In addition, research companies may require researchers to outsource the study to the company or do not authorize researchers to passage and expand the PDX tissue in mice independently. In 2013, several European and US Institutions started the EurOPDX consortium (https://www.europdx.eu/), with the goal of building large collections of models to cover cancer heterogeneity and to raise standards in the preclinical setting. The consortium has collected until now (accessed on 9 February 2022) more than 1500 SC and orthotopic PDX models, including 142 OC. Models are accessible for transfer to academic laboratories on a collaborative basis; from October 2018, part of the collection is available for free-of-charge Transnational Access (TA) through the EurOPDX Research Infrastructure.

Interestingly, the Jackson Laboratory and the European Molecular Biology Laboratory-European Bioinformatics Institute (EMBL-EBI) have implemented PDX Finder, a comprehensive open global catalogue of PDX models and their associated datasets (http://www.pdxfinder.org). PDX Finder currently delivers access to information for 4542 PDX models (about 90 EOC, accessed on 11 March 2022) in eight repositories around the world, including NCI’s Patient Derived Model Repository, The Jackson Laboratory’s PDX Resource, members of the EurOPDX Consortium and members of NCI’s PDXNet [131]. Clickable links will allow users to contact the relevant institution for further collaboration/model acquisition.

Overall, PDX model represents an interesting platform for the identification of predictive biomarkers of response as well as for testing the efficacy of new drugs or new therapeutic strategies. PDXs are also valuable tools for generating drug-resistant tumour models and investigate the molecular basis for this resistance.

### Humanized mouse models

Humanized mouse models are generated by the engraftment of human cancer cell line-derived xenografts (CDXs) or patient-derived xenografts (PDXs) into immunodeficient mice harboring also human immune cells. Different models are commonly used in human oncology studies, with each models having their own strengths and limitations; these different experimental approaches have been reviewed in detail in several previous reports [132]. Humanized mice model platforms are available from different companies and include (a) humanized CD34 + (huCD34) mouse models, ideal for long-term oncology studies as they involve stable engraftment of huCD34 + hematopoietic stem cells (HSC), and produce multi-lineage human immune cells; (b) humanized PBMC (huPBMC, human peripheral blood mononuclear cell) mouse models, ideal for short-term tumour studies evaluating compounds for T-cell immune modulation; (c) knock-in humanized mouse models, including humanized CTLA-4 or PD-1 models, to evaluate the anti-tumour response of immune checkpoint inhibitors directed to human targets in preclinical syngeneic tumour models with a fully functional immune system.

A derivative of the NSG mouse, called NSG-SGM3 (NSGS), is commercially available and may be a useful model for EOC research [133]. This triple transgenic model displays the features of the highly immunodeficient NSG mouse in combination with the expression of human IL-3, GM-CSF (granulocyte macrophage colony-stimulating factor, CSF2) and SCF (stem cell factor, KITLG). When engrafted with CD34 + human hematopoietic progenitor cells, the NSG-SGM3 mice display increased haematopoietic stem cells, B cells, CD33 + myeloid cells, CD3 + T cells, CD4 + T helper cells, and CD8 + T cytotoxic cells. This validated platform support robust tumour growth and can be used for efficacy testing of novel immunotherapies targeting T cells and myeloid cells. This is particularly interesting when considering that the immunosuppressive myeloid cells TAM represent the most abundant host cell population within tumour stroma in EOC and have been shown to drive cancer cells toward a chemoresistant phenotype [48, 134].

In the context of EOC research, Bankert and colleagues [135] published results from an interesting study reporting a simple and reproducible system in which the tumour and tumour stroma were successfully engrafted by injecting tumour cell aggregates derived from fresh ovarian tumour biopsies (including tumour cells, and tumour-associated lymphocytes and fibroblasts), IP into NSG mice. The tumour-derived cell suspensions (from the fresh solid tumour tissue disruption) contained CD45 + leukocytes, cytokeratin-positive cells, and trichrome-positive collagen, which is produced by fibroblasts. This model that recapitulates tumour progression, ascites formation and metastasis as observed in patients, was utilized to evaluate human IL-12 loaded liposomes as a potential immunotherapy for EOC. Later, Chang and colleagues [136] using a humanized mouse model demonstrated that anti-CCR4 monoclonal antibody could restore anti-OC immunity through modulation of Treg activity. More recently, Gitto and colleagues [137] validated an autologous humanized tumour-infiltrating lymphocytes (TIL)/PDX platform for assessing patient-specific T-cell response to immunotherapy and testing immune modulating agents and combination strategies in vivo. The autologous model platform, as that proposed by Gitto et al. [137], has a high translational value, although it is particularly challenging, since the development of each model depends upon the availability of patient tumour tissue and effective TIL expansion.

### GEMMs

Genetically engineered mouse models (GEMMs) of EOC represent excellent preclinical models for studying disease prevention, early detection, and therapy, and have been developed to resemble different molecular phenotypes and histotypes [138, 139]. Certainly, the most effort has gone into the development of GEMMs for HGSOC (reviewed in detail in [138]), although controversies about its cellular origin [140] have made difficult to establish robust models. Now, it is increasingly accepted that both the FTE and the OSE can give rise to HGSOC [19, 20], even if by introducing the same genetic alterations in OSE or FTE, Zhang and colleagues [20] demonstrated that the resulting tumors differed in inter-tumour heterogeneity, molecular pathogenesis, biology, and drug response. The development of HGSOC GEMMs, fully recapitulating early alterations and disease progression seen in patients, still represents an important issue.

Connolly and colleagues [141] at Fox Chase Cancer were among the first who developed a spontaneous transgenic mouse model of EOC by expressing the oncogenic early region of SV40 under the transcriptional control of the Mullerian inhibiting substance type II receptor gene promoter (MISIIR). SV40 Tag binds to and functionally inactivates p53 and Rb [142], which are frequently mutated in human ovarian cancer [143]. Transgenic mice developed, in approximately 50% of cases, bilateral poorly differentiated carcinomas with metastases and ascites; cell lines derived from the ascites (MOVCAR) exhibited the features of EOC and were tumorigenic in immunocompromised mice [141]. Subsequently, to bypass early onset of OC and the lack of fertility, they generated a stable transgenic line of mice, TgMISIIR-TAg-DR26, from an affected male transgenic founder (DR26). In this model, female offspring developed bilateral ovarian carcinomas with 100% penetrance, exhibiting morphology/rapid growth rate similar to human high-grade serous OCs [144]. Notably, these are the only GEMMs that develop spontaneous tumour with pathological features of serous EOC [141, 144]. Finally, the same group isolated individual transgenic lines of non-tumour prone C57BL/6 TgMISIIR-Tag transgenic mice to be used as syngeneic immunocompetent hosts for allografted TAg expressing MOVCAR cells, isolated from tumour bearing C57BL/6 TgMISIIR-TAg-DR26 mice [145]. Orthotopic/IP implantation of MOVCAR cells in TgMISIIR-TAg-Low mice resulted in the development of disseminated peritoneal tumors, resembling to human HGSOC [145].

Later, EOC GEMMs have been developed using specific promoters (e.g., *Pax8* and *Ovgp1*) to drive the inducible expression of Cre-mediated recombination of floxed target alleles in OSE or FTE of engineered mice. This Cre-loxP system allows the conditional knock-in and knock-out of tumour suppressor and/or oncogenes, such as *Trp53*, *Rb1*, *Myc*, *Akt*, *Pik3ca*, *Pten,* and *Arid1a*, considered the major driver genes promoting cancer progression in different EOC. To achieve a spatio-temporal genetic alterations, in some GEMMs, the specific promoter controls the expression of Cre recombinase regulated by tamoxifene or tetracycline [146–148].

Alternatively, a replication-deficient adenovirus altered to express Cre under the control of the CMV promoter (AdCre) has been delivered directly into the space between the ovary and the ovarian bursal membrane to mediate inactivation of floxed genes in engineered mice [149]. Although this approach allowed overcoming the difficulties of identifying specific promoters, it presents some limitations including technical problems associated with IB injection of the adenovirus encoding Cre recombinase. The AdCre based-models show a lower penetrance when compared to promoter-driven models.

Although the benefit of using GEMMs, their establishment requires extensive and time-consuming breeding programs. With the advances in gene editing technologies, such as CRISPR/Cas9, new and much more rapid GEMMs have been developed at a significantly lower cost compared with traditional breading protocols. Recently, two different studies [150, 151] reported a novel strategy to generate somatic EOC mouse models using a combination of in vivo electroporation (EPO) and CRISPR-Cas9-mediated genome editing. Different combination of tumour suppressor/oncogenes (i.e., *Brca1*, *Trp53*, *Pten*, *Lkb1*, Rb, and *Myc*) resulted in successfully generation of HGSOC, showing to be a high flexible and powerful tool. Table 1 summarizes some models developed for different EOC subtypes [152–162]. Notably, the altered genes are important to determine GEMMs histotypes, independently of the method used (i.e., driven-promoter, adenovirus, and electroporation).

**Table 1:** Selection of GEMMs developed for epithelial ovarian cancer research

Cancer histology	Altered genes^a^	GEMMs’ strategy	References
OC	*Trp53;c-Myc;K-ras;Akt*	RCAS	Orsulic et al. [152]
EOC	*Trp53;Rb1*	AdCre	Flesken-Nikitin et al. [149]
SOC	*Trp53;Brca1;c-Myc*	RCAS/Cre	Xing and Orsulic [154]
SOC	*Pten;Pik3ca*	AdCre	Kinross et al. [158]
LGSOC	*Pten;Kras*	Cre driven by *Amhr2* promoter	Mullany et al. [156]
MOC; LGSOC; SOC	*Pten;Kras;Trp53*	Cre driven by *Amhr2* promoter	Ren et al. [161]
HGSOC	*Dicer1;Pten*	Cre driven by *Amhr2* promoter	Kim et al. [157]
HGSOC	*Trp53;Rb;Brca1;Brca2*	AdCre	Szabova et al. [159]
HGSOC	*Trp53;Brca1;Brca2;Pten*	Cre driven by *Pax8-Tet* promoter	Perets et al. [146]
HGSOC	*Trp53;Brca1;Rb1;Nf1;Pten*	Cre driven by *Ovgp1-TAM* promoter	Zhai et al. [148]
HGSOC	*Trp53;Rb*	AdCre	Zhang et al. [20]
HGSOC	*Brca1;Tp53;Pten;Lkb1*	CRISPR-Cas9 Electroporation	Teng et al. [150]
HGSOC	*Trp53;Pten;Rb1;Myc*	CRISPR-Cas9 Electroporation	Paffenholz et al. [151]
EnOC	*Pten;K-ras*	AdCre	Dinulescu et al. [153]
EnOC	*Apc;Pten*	AdCre	Wu et al. [155] Wu et al. [147]
EnOC	*Pten;Arid1a*	AdCre	Guan et al. [160]
EnOC	*Arid1a;Pten;Apc*	AdCre	Zhai et al. [162]
EnOC	*Apc;Pten*	Cre driven by *Ovgp1-TAM* promoter	Wu et al. [147]

^a^Different gene combinations have been tested. *GEMMs* genetically engineered mouse models, *HGSOC* high-grade serous ovarian carcinoma, *LGSOC* low-grade serous ovarian carcinoma, *MOC* mucinous ovarian carcinoma, *SOC* serous ovarian carcinoma, *EnOC* endometrioid ovarian cancer, *EOC* epithelial ovarian carcinoma, *OC* ovarian carcinoma, *RCAS* Replication-Competent ASLV long terminal repeat (LTR) with a Splice acceptor, *AdCre* replication-deficient adenovirus altered to express Cre under the control of the CMV promoter, *AMHR2* Anti-Mullerian Hormone Receptor Type 2, *CRISPR/Cas9* clustered regularly interspaced short palindromic repeats/CRISPR-associated protein 9, *Ovgp1* Oviductal Glycoprotein 1, *Pax8* Paired box gene 8, *TAM* tamoxifen, *TET* tetracycline

Overall, GEMMs allow studying tumour initiation and progression as well as exploring novel therapeutic strategies in immunocompetent and genetically defined mice. Major problems linked to GEMMs are their complex breeding programs and, more importantly, their mixed backgrounds which precludes the development of tumour cell lines that can be used for syngeneic studies, ultimately making them unsuitable for studies of tumour immunity and immunotherapy [146, 148, 157]. Therefore, a syngeneic transplantable model with appropriate mutations would represent a more reliable model*.* As already stated before, the most frequently used murine cell line ID8 does not retain the typical mutations and copy-number alterations that define human HGSOC. Besides the genetic engineering of the ID8 model discussed above [99], an interesting approach has been proposed by Maniati and colleagues [163] who recently characterized the TME of six orthotopic, transplantable syngeneic murine HGSOC lines established from GEMMs backcrossed onto B6 background [146] and GEMMs generated by adenovirus transduction [159]. Interestingly, they showed that many of the biomechanical, cellular, and molecular features of human HGSOC were reproduced in the murine tumors, with significant correlations in mRNA expression profiles, innate and adaptive immune responses, tissue modulus, and matrisome components [163].

### Laying hen model

The only non-human animal that spontaneously develops ovarian cancer with a high prevalence is the laying hen (*Gallus domesticus*). Tumors developed by laying hen are remarkably similar to human disease with an incidence ten-fold higher than in women [164]. Barua and colleagues have reported that histological types as well as stages of EOC in hens are similar to humans [165]. Likewise, the risk of EOC development is highly correlated with age and number of ovulation [164]. Therefore, this model provides the opportunity to study risk factors for EOC as well as tumour initiation, progression, histological origin, and therapy response. In addition, it represents a valuable tool for preclinical testing of cancer therapy. However, the cellular origin of EOC in hens is controversial as observed in humans, being both the ovary and the oviduct involved at the time of cancer diagnosis. Recently, Paris and colleagues have developed a preclinical model of spontaneous EOC, particularly HGSOC, originated from oviductal fimbria [166]. This study, not only highlighted the similarities in term of histology and molecular markers between malignancies developed in hens and women, but, most importantly, offered the possibility to study different aspects of spontaneous HGSOC in women, including its early detection [166].

Overall, despite anatomical and physiological differences, the laying hen model offers benefits compared to murine xenograft models and GEMMs regarding the etiology and pathogenesis of EOC.

### Zebrafish model

In recent years, the zebrafish (*Danio rerio*) has emerged as an attractive alternative to mouse in cancer research representing an efficient platform for investigating cancer and cancer therapeutics. The strengths of this model are the high fecundity, rapid external development, as well as easy, low-cost maintenance [167, 168]. Both embryos and adult zebrafish can be used for drug screening, although for embryos, the drug administration in their water is easier. About 70% of human genes have at least one zebrafish orthologue, and therefore, zebrafish cancer models, similar to human cancer, can be easily produced manipulating zebrafish genetics [169]. To overcome some drawbacks related to the genetically engineered models, as difficulties in controlling each step of disease, the technique of cancer cell transplantation into zebrafish embryos and adults has been used (reviewed in [170]). Among the advantages for using a zebrafish xenograft models, Kirchberger counts the lack of functional adaptive immune system in zebrafish larvae within the first 4 weeks after fertilization that prevents rejection of xenografts [170].

Moreover, zebrafish embryos and the adult Casper zebrafish line are excellent model organisms to image tumour development, metastasis, and microenvironmental interactions due to their optical transparency [167]. Using EOC cell line-bearing zebrafish embryos, zebrafish xenotransplantation models have been developed for assessing cancer progression or testing anticancer compounds [171, 172]. In more detail, Latifi and colleagues have injected OVCA433 cells [either untreated or treated with cisplatin or cisplatin + U0126 (a highly selective MEK1 and MEK2 inhibitor)], in zebrafish embryos to evaluate cell migration after 48 h [171]. Later, Wang and colleagues [172] used the same model to evaluate proliferation and metastasis of A2780 cells treated with different compounds. These studies suggest that zebrafish can be considered a valuable preclinical model of EOC complementing long-term mouse models. Interestingly, PDX using zebrafish as “avatar” for developing precision medicine strategies have been recently carried out both for patient tumour cells and tissues. However, these latter models are currently under development in EOC [173]. Importantly, some weaknesses limit the use of zebrafish for cancer research. First, the presence of an underdeveloped adaptive immune system in larvae is, on one side, a benefit in term of no rejection of xenografts but, on the other side, an obstacle for studying fully functional TME. Besides, physiological conditions are different between zebrafish and human, and especially, the temperature represents a critical factor (as reviewed in [174]).

## Conclusions

Development of experimental models for EOC research presents significant scientific challenges. Essentially, it remains extremely difficult identifying high-quality tools with which recapitulate the molecular mechanisms underpinning the pathophysiology and the therapy-resistant recurrent disease to which patients ultimate succumb.

**Fig. 2:**
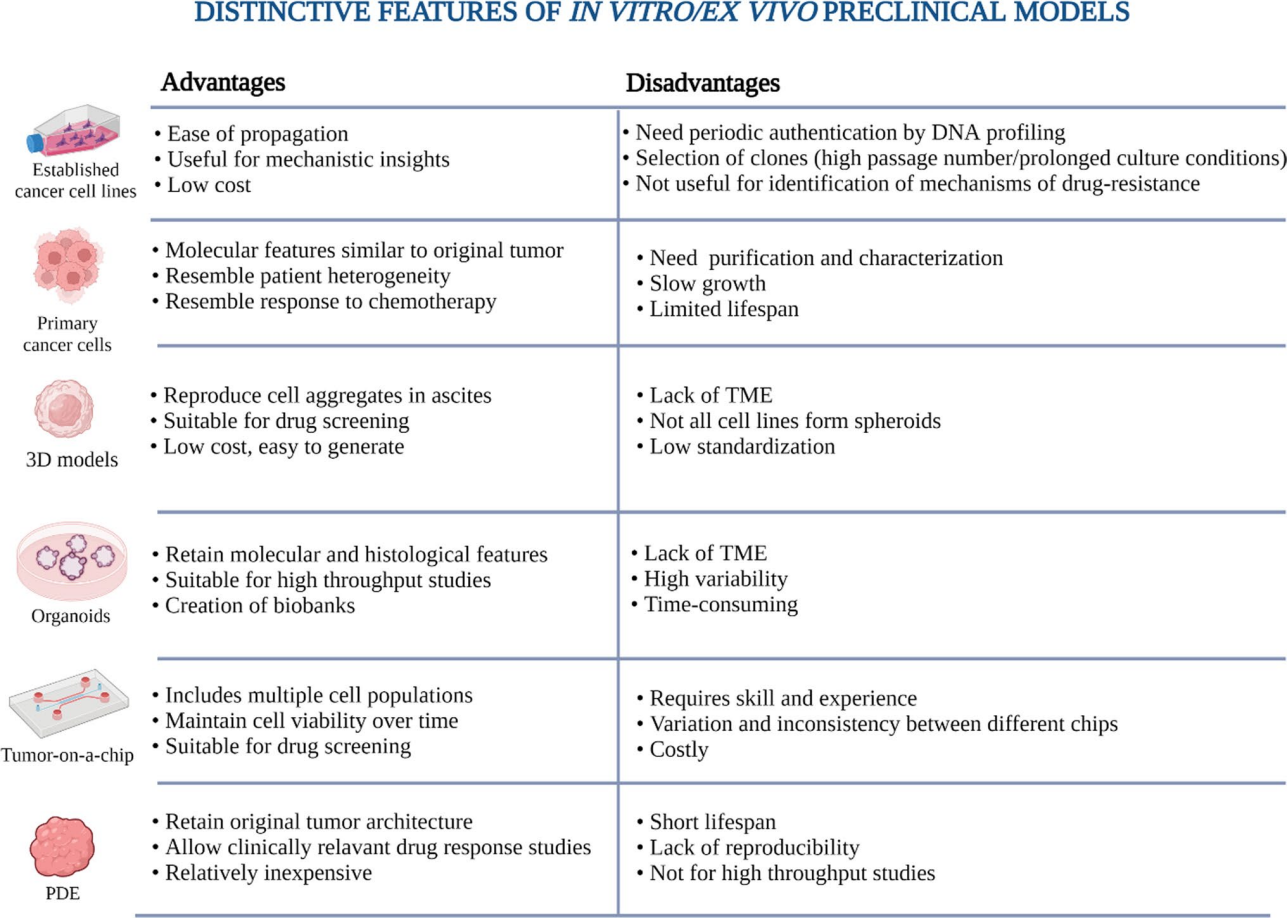
Distinctive features of in vitro preclinical models. Features are divided into model-favoring characteristics and limiting factors. *PDE* patient-derived explants, *TME* tumour microenvironment. This figure was created with BioRender.com

Established cell lines have been a fundamentally discovery tool in EOC, although an important matter that previous research has brought to light is that the most highly used cell lines are actually not representative of HGSOC, the most prevalent EOC subtype. Therefore, appropriate choice of cell lines for the different histotypes of EOC along with routine analysis of cell line authenticity are the first steps to increase the translational value of cell line studies in preclinical research on EOC. Besides, our and literature data have also demonstrated that established EOC cell lines, although easier to work with than primary cells, may not be recommended to understand determinants of sensitivity to current therapies or to translate novel therapies into the clinics [6, 175]. These models, however, under the right conditions and with the appropriate controls, retain their utility in mechanistic work at the protein and pathway level (Fig. 2).

On the other hand, primary tumour cell cultures more closely resemble the patient situation, and therefore, they represent a more experimentally accurate model for the reproduction of cancer in in vitro systems. Due to these unique features, they are a valuable experimental tool during preclinical studies of drug resistance mechanisms, as we also recently demonstrated [175]. However, the need of characterization for determining origin and purity, the limited lifespan of days or weeks, and the slow growth, constitute important limitations to their use (Fig. 2).

Irrespective of their origin, 2D cell cultures cannot faithfully reproduce the real tumour microenvironment. Three-dimensional cell cultures, mirroring the physical and biochemical features of a solid tumour mass, show promise to bridge the gap between traditional 2D cell culture and in vivo animal models. Indeed, various studies have demonstrated that cancer cell lines grown in 2D and 3D culture often exhibit different gene/protein expression profiles, being the 3D system profiles more similar to those of the original cancer tissue [43]. Importantly, 3D cell culture has become one of the top methods of choice in drug discovery, being also better predictors of in vivo drug responses (Fig. 2). In this context, organoid-based 3D culture methodology, offering important advantages to spheroids, has revealed a great potential as a physiologically relevant in vitro platform to elucidate EOC biology and to predict in vivo patient responses [59] (Fig. 2).

The biological translation of these in vitro systems is limited by the lack of the tumour-associated microenvironment, and therefore, they are not entirely suitable to capture the integrated biological properties of the native tumour. Approaches to model the ovarian TME, including co-culture with different cell types, such as immune cells, endothelial cells, and stromal cells, allowing a closer reproduction of the in vivo conditions, may offer major opportunities to improve translatability. In this context, the emerging tumour-on-a-chip platforms, integrating 3D cell culture, microfluidic technology, and tissue engineering hold great potential for being exploited as an alternative to in vivo animal studies, possibly accomplishing the international priority of the 3R principles, for Replacement, Reduction, and Refinement of animals in research (Fig. 2).

The above discussed models suffer from the drawback of not contextually preserving human tumour architecture. By contrast, ex vivo models, including tissue slices or patient-derived EOC explants, without any requirement for deconstruction or reconstruction, offer a high content in vitro experimental platform, which can be successfully used both for exploring individualized treatment therapies and for studying cancer biology. On the other hand, these models lack a functional vasculature, have a short lifespan, and are expensive and difficult to maintain, and they are not suitable for high-throughput approaches (Fig. 2).

**Fig. 3:**
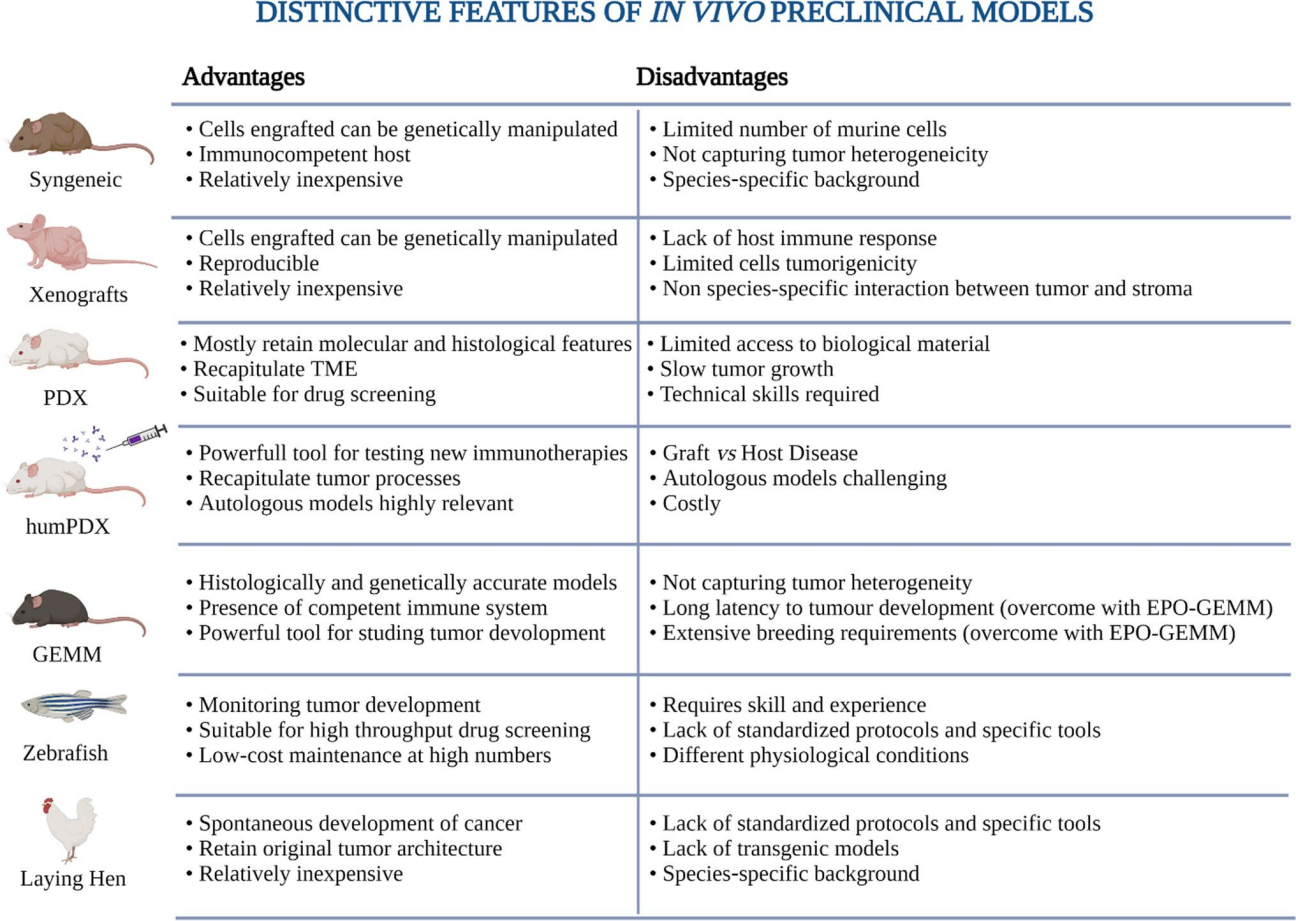
Distinctive features of in vivo preclinical models. Features are divided into model-favoring characteristics and limiting factors. *PDX* patient-derived xenograft, *humPDX* humanized PDX, *GEMMs* genetically engineered mouse model, *EPO-GEMM *in vivo organ electroporation approach, *TME* tumour microenvironment. This figure was created with BioRender.com

On the whole, in vitro and ex vivo preclinical approaches are all limited by their inability to reproduce the interplay among multiple systems, with significant disadvantages in the explorations of drug pharmacokinetics and pharmacodynamics. Therefore, in vivo models still keep a prominent place in EOC research (Fig. 3). In spite of this, translational EOC research field still lacks an animal model that is both robust and widely accessible, being well recognized that each model has its own advantages and disadvantages. The biological/clinical value of the different experimental approaches should be determined on a case-by-case basis to address specific issues, bearing in mind that the use of multiple models can increase confidence in study results. PDXS and GEMMS are the preferred in vivo research platforms for EOC, due to the limitations associated with cell line-derived xenografts [176]. Patient-derived xenografts largely maintain the characteristics of the patients’ original tumour including histology, mutational status, gene expression, and clinical behavior, while remaining generally stable throughout propagation, with marginal genetic drift at the time of PDX initiation and adaptation to mouse host. Therefore, PDX studies have proven effective in identifying clinically relevant biomarkers and in predicting human outcomes and response to conventional drugs in EOC therapy (Fig. 3). Importantly, different preclinical studies have confirmed the power of humanized mouse models for testing novel EOC immunotherapies, particularly in the emerging PDX platforms involving the engraftment of both tumour explants and immune cells from the same patient to establish a human immune system in the mouse host (Fig. 3). Their use is, however, currently limited by costs and relatively long and unpredictable times for establishing test animals. On the other hand, transgenic murine models may recapitulate a specific cancer pathway, but fail to capture the true heterogeneity that is characteristic of human tumors. Other limitations include an extensive and costly breeding program and a variable latent period for tumour formation (up to 12 months in some instances) (Fig. 3).

In conclusion, no preclinical model can fully recapitulate the complexity of EOC within patients and their interindividual variability in drug response. A reasonable approach is to use a combination of vitro, ex vivo*,* and in vivo experimental platforms to improve the predictive power of experimental systems, hopefully enhancing the impact of cancer research in EOC.

## Supplementary Information

Below is the link to the electronic supplementary material.

Supplementary file1 (XLSX 15 KB)

## Data Availability

Not applicable.

## Abbreviations

2D: Two-dimensional
3D: Three-dimensional
3R: Replacement, Reduction and Refinement
AdCre: A replication-deficient adenovirus altered to express Cre under the control of the CMV promoter
Akt: Thymoma viral proto-oncogene 1
AMHR2: Anti-Mullerian hormone receptor type 2
Apc: Adenomatous Polyposis Coli
Arid1a: AT-rich interactive domain 1A
ASC: Adult stem cells
ATCC: American Type Culture Collection
BOT: Borderline ovarian tumors
BRAF: B-Raf proto-oncogene, serine/threonine kinase
BRCA1: Breast Cancer gene 1
BRCA2: Breast Cancer gene 2
BRCAMUT: BRCA1/2 mutations
CAN: Copy-number alteration
CBA: CellBank Australia
CCC: Clear-cell carcinoma
CCLE: Cancer Cell Line Encyclopedia
CCNE1: Cyclin E1
CCR4: C–C chemokine receptor type 4
CDXs: Cancer cell line-derived xenografts
CLASTR: Cellosaurus STR similarity search tool
CNV: Copy-number variation
COSP: Calculator for Ovarian Subtype Prediction
CRISPR/Cas9: Clustered regularly interspaced short palindromic repeats/CRISPR-associated protein 9
CSC: Cancer stem cells
DEGs: Differentially expressed genes
DMBA: 7,12‑Dimethylbenz[*a*]anthracene
DSMZ: Deutschen Sammlung von Mikroorganismen und Zellkulfuren
EBV: Epstein–Barr virus
ECACC: European Collection of Cell Cultures
ECM: Extracellular matrix
EGF: Epidermal growth factor
EMBL-EBI: European Molecular Biology Laboratory-European Bioinformatics Institute
EnOC: Endometrioid ovarian carcinoma
EOC: Epithelial ovarian cancer
EPO: In vivo electroporation
ESC: Embryonic stem cells
FTE: Fallopian tube epithelial
GEMMs: Genetically engineered mouse models
GM-CSF: Granulocyte macrophage colony-stimulating factor
HDRA: Histoculture drug response assay
HGSOC: High-grade serous ovarian carcinoma
HR: Homologous recombination
HSC: Hematopoietic stem cells
hTERT: Human telomerase reverse transcriptase
HPV: Human Papilloma Virus
huPBMC: Human peripheral blood mononuclear cell
IB: Intrabursal
IDS: Interval debulking surgery
IP: Intraperitoneal
iPSC: Induced pluripotent stem cells
IVIS: In Vivo Imaging System
JCRB: Japanese Collection of Research Bioresources
KITLG: KIT ligand
KRAS: Kirsten rat sarcoma virus
LGSOC: Low-grade serous ovarian carcinoma
Lkb1: Liver kinase B1
MCAs: Multicellular aggregates
MEK1: MAP (Mitogen-Activated Protein) Kinase/ERK (Extracellular Signal-Regulated Kinase) Kinase 1
MEK2: MAP (Mitogen-Activated Protein) Kinase/ERK (Extracellular Signal-Regulated Kinase) Kinase 2
MFP: Mammary fat pad
MISIIR: Mullerian inhibiting substance type II receptor
MOC: Mucinous ovarian carcinoma
MOSE: Mouse ovarian surface epithelial
MOVCAR: Murine ovarian carcinoma
MRI: Magnetic resonance imaging
MTT: 3-(4,5-Dimethylthiazol-2-yl)-2,5-diphenyl-2H-tetrazolium bromide
MYC: MYC proto-oncogene, bHLH transcription factor
Myc: Myelocytomatosis oncogene
NACT: Neoadjuvant chemotherapy
Nf1: Neurofibromin 1
NOD: Non-obese diabetic
NRG: NOD-Rag1-/-IL2RgammaC-null
NSG: NOD/SCID/IL2Rγ null
NSGS: NSG-SGM3
OC: Ovarian cancer
OSE: Ovarian surface epithelial
OTME: Ovarian tumour microenvironment
Ovgp1: Oviductal glycoprotein 1
PARP: Poly (ADP-ribose) polymerase
Pax8: Paired box gene 8
PD-1: Programmed cell death 1
PDE: Patient-derived explant
PD-L1: Programmed cell death ligand 1
PDO: Patients-derived organoid
PDX: Patient-derived xenograft
PDS: Primary debulking surgery
PIK3CA: Phosphatidylinositol-4,5-Bisphosphate 3-Kinase Catalytic Subunit Alpha
Pten: Phosphatase and tensin homolog
Rb: Retinoblastoma protein
RNA: Ribonucleic acid
SC: Subcutaneous
SCF: Stem cell factor
SCID: Severe combined immunodeficiency
SRC: Subrenal capsule
STR: Short tandem repeat
SV40: Simian virus 40
TA: Transnational Access
TAM: Tumor-associated macrophages
TIL: Tumor infiltrating lymphocytes
TME: Tumor microenvironment
TP53: Tumor protein p53
Trp53: Transformation-related protein 53
UKCTOCS: UK Collaborative Trial of Ovarian Cancer Screening
US: Ultrasound
VAF: Variant allele frequency
